# LIVING WITH DEMENTIA: EXPERIENCES OF LGBTQ PEOPLE WITH DEMENTIA AND SIGNIFICANT OTHERS

**DOI:** 10.1093/geroni/igad104.0255

**Published:** 2023-12-21

**Authors:** Anna Siverskog, Linn Sandberg

**Affiliations:** Södertörn University, Stockholm, Stockholms Lan, Sweden; Södertörn university, Huddinge, Stockholms Lan, Sweden

## Abstract

Dementia is commonly conceptualized in biomedical terms as a deterioration of brain function and a subsequent loss of self. However, dementia scholars have increasingly underscored how the self of the person with dementia may be maintained through social interactions in their everyday life. This may pose specific challenges for LGBTQ people who may not be out in care settings due to fear of discrimination and whose chosen family may not be recognized by formal care providers. This paper presents preliminary findings from a qualitative study conducted in Sweden with LGBT individuals living with dementia. The project builds on policy analysis as well as semi-structured interviews with LGBTQ people with dementia, people significant to them such as partners, family and friends, and interviews with frontline care staff and managers within dementia care. The thematic analysis illustrates how experiences of living with dementia as an LGBTQ person vary depending on the progression and type of dementia, the individuals’ social support network, how open one is with their sexual orientation or gender identity, living at home or in a dementia care facility, socioeconomics, and other factors. In line with how other ageing scholars have pointed to the potentiality of queer theory to move beyond framing dementia as pathology and decline, concepts of queer time and queer failure are used here to understand the results in ways that disrupt notions of self, temporalities, normativity, subjectivity and what it means to age successfully.

